# Metagenomic sequencing detects viruses and bacteria in a cross-sectional clinical cohort of undifferentiated febrile illness in Nigeria

**DOI:** 10.1128/msphere.00157-26

**Published:** 2026-08-12

**Authors:** Grace J. Vaziri, Julia C. Pritchard, Jillian I. Howard, Grace E. Stamm, David H. O'Connor, Christina M. Newman, Matthew T. Aliota, Asabe Dzikwi-Emennaa

**Affiliations:** 1 Department of Veterinary and Biomedical Sciences, University of Minnesota, Twin Cities 5635, Minneapolis, Minnesota, USA; 2 Department of Pathology and Laboratory Medicine, University of Wisconsin-Madison 189586, Madison, Wisconsin, USA; 3 Department of Veterinary Public Health and Preventive Medicine, University of Jos 100570, Jos, Plateau, Nigeria; Universitatsklinikum Hamburg-Eppendorf, Hamburg, Germany

**Keywords:** metagenomics, undifferentiated febrile illness, virus, etiology

## Abstract

Molecular and microscopy-based diagnostic capacity is often insufficient or unavailable in places where infectious disease burdens are highest, such as in West Africa. Rapid diagnostic testing (RDT) can provide quick and affordable diagnoses of common infections but is an imperfect solution due to limitations around detecting and dealing with false-negative and false-positive results. An alternative to RDT is unbiased metagenomic sequencing for pathogen surveillance. Here, we present data from unbiased metagenomic sequencing used to identify causes of undiagnosed febrile illness in Jos, Plateau State, Nigeria. Proof of concept for this approach has been demonstrated by several groups who have identified epidemic and endemic viral diseases like Lassa fever, yellow fever, and chikungunya. We show that unbiased deep sequencing and metagenomic analysis can be used to identify RNA viruses in clinical samples. We sequenced RNA from sera of patients (*n* = 343), many of whom were acutely febrile (76%), in a survey of clinics in Jos. We detected five human-infecting viruses in 39 (11 %) specimens. Among these were hepatitis B virus, human pegivirus, and several anelloviruses. While most of the viruses identified are unlikely to cause clinical symptoms in the patients we sampled, their presence demonstrates the validity of our approach. Additionally, our sequencing data allowed us to identify genetic material from potentially pathogenic bacteria, another possible etiological agent of febrile illness.

**IMPORTANCE:**

In low-resource areas, fevers due to infectious pathogens are a major source of illness, but tools for detecting and identifying such pathogens are often limited. Unbiased approaches for identifying genetic material from all potentially infectious organisms in a sample represent an opportunity for discovering sources of fever. Metagenomic sequencing can improve insight into pathogen landscapes in low-resource settings, potentially providing early detection of disease outbreaks. However, unbiased metagenomic sequencing (mNGS) is no panacea; it is susceptible to contamination and false positives. We used mNGS to evaluate serum from >300 Nigerian clinic-goers in Jos, Nigeria, most of whom (>70%) had fevers of unknown origin. Our goal was to understand arbovirus prevalence in Jos, Nigeria, and identify the sources of infection not routinely monitored for at clinics. We detected hepatitis B virus, as well as nonpathogenic anelloviruses. Our study provides insight into the utility and limitations of mNGS for pathogen surveillance.

## INTRODUCTION

In low- and middle-income countries, febrile illnesses with unknown etiologies are common, while investigations of potential viral etiologies are rare (1, 2). For example, a review of studies investigating the sources of febrile illnesses noted that only 17% of investigations even considered viral etiologies (2). In Sub-Saharan Africa, molecular diagnostics are limited; hence, rapid diagnostic testing (RDT) is often used to detect common fever-causing infectious agents, including malaria, typhoid, SARS-CoV-2, tuberculosis, and HIV (3–5). The suite of potential causes of febrile illness, however, far exceeds the capacity for RDT and includes fungal, bacterial, protozoal, and viral infections (5). While RDT has been generally considered a success story in Africa, there are limitations, including sensitivity and specificity issues, cross-reactivity leading to misdiagnosis, the evolution of diagnostic resistance, and the inability of these technologies to characterize the infecting agent if a patient tests negative (6).

Failure to detect and identify the true etiology of febrile illness has detrimental effects for both individual patients and public health (7). In complex disease landscapes such as Nigeria, common circulating infectious agents may mask the presence or emergence of cryptic or rare sources of infection (8). As of the writing of this manuscript, an outbreak of Bundibugyo virus disease (caused by a rare strain of Ebola virus) is growing in the Democratic Republic of the Congo. Its spread was likely enhanced because early testing of suspected cases was performed with a Zaire-specific test, producing false negatives that led to costly delays in outbreak response (9). Individual patients without a definitive diagnosis are at greater risk of receiving incorrect (or no) therapeutic interventions. Furthermore, if clinicians are unable to detect the source of febrile illness, they may resort to the presumptive use of antimalarials and antibiotics in a stopgap effort to treat their patients. Inappropriate use of antimicrobial drugs is termed “overtreatment” and can promote the evolution of drug resistance among their intended targets (e.g., the spread of mutations conferring artemisinin resistance in *Plasmodium falciparum* in Cambodia and Rwanda [7, 10, 11]). A study of children under 5 years old in Nigeria documented an overtreatment rate of 83% based on microscopic confirmation of malaria parasitemia (12). Ultimately, failure to detect and identify the sources of febrile illness may stymie the ability of public health workers to implement effective public health interventions and could potentially enhance the progression of infectious disease outbreaks to epidemic levels.

Metagenomic sequencing (mNGS) is increasingly used as a method for detecting the presence of pathogens from both individual-level (i.e., patient [13–15]) and aggregate-level (e.g., air and wastewater [16, 17]) samples. The taxon-agnostic approach of metagenomic sequencing, in which all genetic material (RNA or DNA, depending on study goals) from a sample is sequenced and identified, distinguishes it from targeted approaches, in which samples are assessed for the presence of predetermined pathogens of interest. When causative agents of illness are unknown and potentially heterogeneous across a patient cohort, mNGS can be a powerful tool for identifying the presence of pathogenic microbes. The data reported in this manuscript were collected with an original intent to improve understanding of arthropod-borne virus (arbovirus) circulation in Jos, Nigeria. Arboviruses are not routinely tested for in healthcare facilities in Jos, Nigeria, despite chikungunya virus, dengue virus, yellow fever virus, Zika virus, and other arboviruses that infect humans all occurring in Nigeria (18). We chose to use an unbiased metagenomic approach rather than a targeted approach to cast a wide net that allows us to consider multiple pathogens simultaneously and maximize the likelihood that we could detect potentially rare or unexpected viruses. Additionally, it is critical to continue to ground truth the most beneficial applications of metagenomics in real-world scenarios because the cost and the technical skills needed for data generation and analysis preclude widespread deployment in resource-limited settings. The potential information gained from pathogen-agnostic sequencing approaches is especially enticing in cases where RDT and patient symptomatology yield negative and/or non-specific conclusions. For example, a mNGS study of sera from acutely febrile children in Tanzania identified rotaviruses and enteroviruses (both in family *Picornaviridae*) in >10% of samples; these infectious agents are not traditionally monitored for using RDTs (19). Beyond improving the ability of clinicians and researchers to identify potential fever etiologies, mNGS can detect the presence and circulation of asymptomatic infections. Identifying both asymptomatic infections and infections with relatively nonspecific symptoms is important to minimize cryptic transmission and exposure to susceptible patients for whom infections are likely to be more severe. For example, many arboviral pathogens, such as dengue, Zika, and West Nile viruses, can sometimes result in asymptomatic infections, but in some patients, they can also cause severe pathology (20–22). Early detection of these infections in both asymptomatic and symptomatic patients can improve the efficacy of public health responses.

In this study, we used mNGS to detect RNA from human-infecting pathogens among a group of patients who visited four clinics in Jos, the capital city of Plateau State, Nigeria, in June–October 2023. Jos, Nigeria has a high burden of infectious diseases. Malaria and typhoid fever are common infections among patients from this region and are often assumed to be the cause(s) of febrile illnesses (23). We therefore collected serum from a mixed-sex, mixed-age population of patients that included febrile and afebrile patients. Samples came from patients with confirmed malaria or typhoid fever and from patients with no diagnosis. We report the detection of several common, nonpathogenic anelloviruses, as well as two detections of hepatitis B virus, and reads from 53 bacterial taxa known to infect humans.

## MATERIALS AND METHODS

### Sample collection

**Fig 1:**
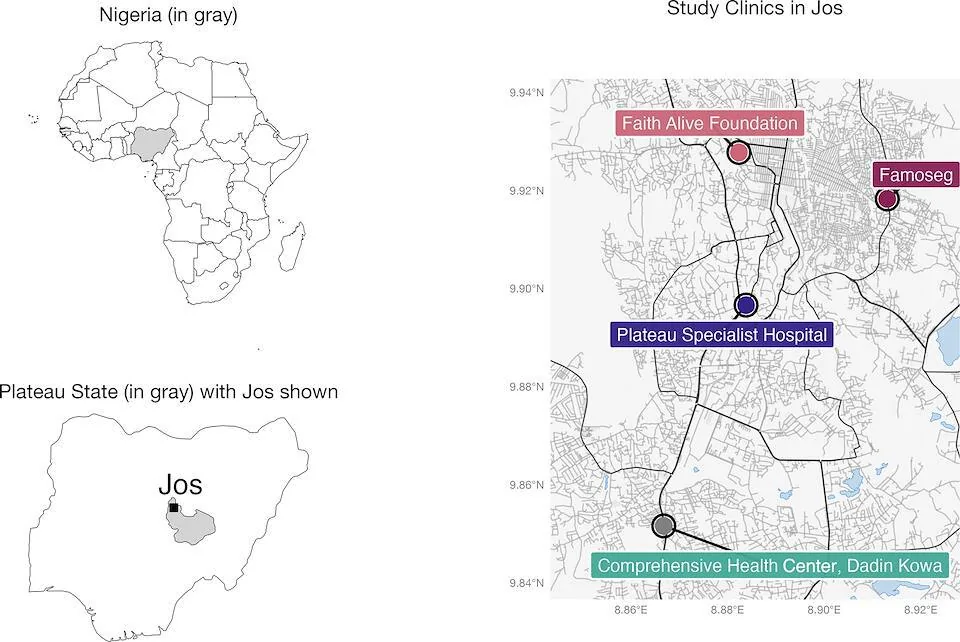
Serum samples were collected from patients who visited four clinics in the city of Jos, Plateau State, Nigeria. Sample collection was unevenly spread across hospitals, with most samples coming from the Faith Alive Foundation and Famoseg clinics.

Samples were collected from four clinics (Fig. 1) during the 2023 rainy season (between late June and late October) in Jos, Plateau State, Nigeria.

Patients whose temperatures were above 37.2°C were categorized as febrile. A subset of patients were administered RDTs for either malaria (*P. falciparum*) or typhoid (Table 1). Data were collected from anonymized patients who agreed to participate in the study at the time of their clinic visit. Socioeconomic, demographic, and symptom-related data were collected by trained interviewers who obtained patient consent prior to reading questions aloud and recording patients’ responses in a standardized form. The serum was separated from blood collected during clinic visits and stored in 2 mL cryotubes at −20°C for storage prior to export. Samples were picked up on 29 January 2024 from the University of Jos and were transported from the Lagos airport to the Frankfurt airport, where they remained until 3 February 2024 due to a strike among airport staff. Dry ice was reported to be replenished during this time, but the courier provided no confirmation of this. Samples were then shipped to New York, USA, where they arrived on 4 February 2024 and remained until 19 February 2024. Dry ice was replenished daily during this period. Although shipping containers contained temperature monitors, all were nonfunctional when they arrived, and requests for temperature reports from the courier were denied. The serum appeared to have remained frozen during transit (evidenced by tubes retaining serum in the bottom of the tube, despite being jostled and arriving upside-down), but we cannot definitively confirm that they remained frozen throughout all transit. Following the receipt of samples in the United States, samples were stored at −80°C until RNA extraction.

**TABLE 1:** A small subset of patients was confirmed via rapid diagnostic tests to have (or to not have) malaria or typhoid infections; however, most were not provided with a diagnosis at the time of sample collection (“not tested”)^*a*^

Diagnosis at time of clinic visit	*N*_all_ (all in initial sample set)	*N*_NVD_ samples that remained after filtering for analysis with NVD)	*N*_CZID_ (samples that remained after filtering for analysis with CZ ID)
Malaria negative and typhoid positive	15	8	7
Malaria negative and typhoid unknown	152	119	111
Malaria positive and typhoid positive	1	1	1
Malaria positive and typhoid unknown	24	19	20
Typhoid negative and malaria unknown	1	1	1
Typhoid positive and malaria unknown	11	7	7
Not tested	282	188	212
Total	**486**	**343**	**359**

^*a*^ Based on method-specific filtering criteria, we retained a slightly different number of samples from patients in each diagnostic category. Total sample size (denominator) for all samples collected, samples retained for NVD analysis, and samples retained for CZID analysis are reported in bold on the bottom row.

### Sample processing

Prior to RNA extraction, the samples were inactivated in a biosafety level three (BSL 3) laboratory with TRIzol LS supplemented with chloroform at 20%, and then, RNA was extracted in batches of 23 samples plus an extraction control at BSL 2 (24). After phase separation, RNA was precipitated with isopropanol, washed with ethanol, and re-suspended in a final volume of 30 µL nuclease-free water. A no-template control sample (water) was extracted each time RNA was extracted from a batch of serum samples to control for contamination in the extraction space or reagents. To avoid confounding effects due to RNA extraction batch, sampling clinic, or time of sample collection, we ensured that clinics were proportionally represented in each extraction batch according to their prevalence in the total sample set and then randomly assigned samples to RNA extraction batches. All RNA was stored at −80°C immediately after extraction until cDNA synthesis.

### cDNA synthesis

Double-stranded cDNA from extracted RNA transcripts was prepared using a modified sequence-independent single-primer amplification (SISPA) approach (25, 26). To reverse transcribe RNA templates, we prepared a 40 pmol/μL working stock of primer A [5′-GTT TCC CAC TGG AGG ATA-(N9)-3′] and added 1 µL of primer A working stock to 4 µL of extracted RNA. We heated this at 65°C for 5 min, followed by 5 min of cooling at 4°C. To perform first strand synthesis, we added 2 µL 5× RT buffer, 1 µL 10 mM dNTPs, 1 µL nuclease-free water, 0.5 µL 0.1 M DTT, and 0.5 µL SuperScript IV RT enzyme to each sample (all reagents from Invitrogen 18091200) and incubated for 10 min at 42°C. We performed second strand synthesis by adding 1 µL of Sequenase reaction buffer, 0.15 µL Sequenase DNA polymerase (Applied Biosystems 70775Y200UN), and 3.85 µL nuclease-free water to each reaction and incubating at 37°C for 8 min. After incubation, we added 0.45 µL Sequenase dilution buffer and 0.15 µL Sequenase DNA polymerase (Applied Biosystems 70775Y200UN) to each sample and incubated again for 8 min at 37°C. Finally, we amplified 5 µL of template cDNA by adding 2 µL of primer B (5′-GTT TCC CAC TGG AGG ATA-3′), 25 µL LongAmp Taq 2× master mix (NEB M0287S), and 18 µL nuclease-free water and running PCR with the following conditions: 94°C for 30 s, followed by 30 cycles of 94°C for 15 s, 47°C for 20 s, and 65°C for 2 min, followed by 65°C for 10 min. We prepared a negative control during the cDNA synthesis step for each batch of cDNA synthesis using nuclease-free water instead of an RNA template. Following cDNA synthesis, we quantified 2 µL of each cDNA product using the Qubit dsDNA HS Assay Kit (Invitrogen Q32851).

### Nanopore library preparation

We prepared libraries for nanopore sequencing using native barcoding (Oxford Nanopore SQK-NBD114-24 and SQK-NBD114-96) (https://nanoporetech.com/document/ligation-sequencing-gdna-native-barcoding-v14-sqk-nbd114-24?device=MinION). Briefly, we followed the protocol provided by Oxford Nanopore to take forward up to 400 ng of cDNA (or 11 µL of cDNA template if cDNA concentration was insufficient) into DNA repair and end preparation. After end preparation, we ligated native barcodes, pooled barcoded samples, and performed adaptor ligation and cleanup. Following adaptor ligation, we cleaned up the library with AMPureXP beads and washed the beads twice with 125 µL short fragment buffer. In addition to patient samples and negative controls from previous steps (RNA extraction and cDNA synthesis), each of our final sequencing libraries contained a negative control (nuclease-free water) on which we performed preparation and barcoding to control for contamination in the library preparation reagents and spaces. Additionally, we extracted RNA, synthesized cDNA, and sequenced at least two positive controls with every library. The first of these positive controls was a Powassan virus (POWV) isolate used in the Aliota (MTA) laboratory and included to ensure that our sequencing approach could successfully handle viral RNA. The second of the positive controls was synthetic RNA spike-ins (SRSIs) containing 10,000 gene copies of a foreign control sequence (27). We included a unique SRSI (sequences in Data S1) with each library we sequenced and used these sequences to track cross-contamination rates between sequencing runs. We eluted the final library in 15 µL of elution buffer and quantified average fragment length and concentration using an Agilent Bioanalyzer. Using the average fragment length and concentration of our final library, we calculated the volume of library that comprised 300 fmol to carry forward to sequencing. Previous experience using nanopore sequencing for mNGS applications indicated that recommended loading concentrations from the product manual are too low for libraries, as they generally comprise short fragments (about 490 bp), leading to underloading of flow cells and reduced data yields.

### Bioinformatic methods

We basecalled all libraries using the high-accuracy basecalling model (dna_r10.4.1_e8.2_400bps_hac@v5.0.0). After demultiplexing libraries and discarding those with fewer than 10,000 raw sequencing reads, we processed samples using the NVD (v. 2.0) workflow (https://github.com/dhoconno/nvd). The NVD workflow performs initial classification of human virus sequences with the NCBI STAT tool (28). Following STAT classification, the workflow uses a two-step BLAST verification, first performing a rapid sequence similarity search with megablast, then using the more sensitive blastn tool to align and classify contigs that were unclassified with megablast. The result of this two-step classification procedure was a list of all megablast hits for any contig that BLASTed to a viral reference sequence, and up to five blastn hits for any contig that blasted to a viral sequence. To generate a one-to-one list of contigs and taxon assignments (BLAST hits), we selected the single best match based on E-score for each sample/contig combination. We imported this list into R (v. 4.2.2) along with a list of read counts per contig to generate a taxon-by-sample matrix filled with read counts (i.e., an OTU table) with phyloseq v. 1.44.0 (29). Next, we used the “isContaminant()” function, a statistical decontamination algorithm, from the decontam (v. 1.18.0) package (30) with the “combination” method enabled and specifying the sequencing library as a batch variable. After removing reads identified by decontam as statistical contaminants, we removed reads from all non-viral taxa that remained in the data set. We also consolidated reads from viruses that were uninformatively listed as being separate (e.g., reads from Human pegivirus and *Pegivirus hominis* were combined). While NVD is specifically designed to detect viruses that can infect humans, the method of taxonomic classification (megablast + blastn) combined with subsequent selection of a single best taxonomic assignment led to non-viral reads being included in the data set. This occurred when a contig did not blast to viral sequences unanimously and a non-viral sequence had the lowest E-value of all blast hits. Finally, we manually inspected the remaining taxa to verify they met the following criteria: (i) are known to infect humans, (ii) are not possible lab contaminants, and (iii) have at least five reads.

In addition to our virus-focused analysis, we used two methods of taxon-agnostic sequence profiling: GOTTCHA2 (v. 2-2.1.10) and CZ ID (formerly IDseq). The GOTTCHA2 pipeline is a gene-independent taxonomic profiling algorithm incorporated into the larger NVD workflow. GOTTCHA2 classifies bacterial and eukaryotic reads in addition to viral (31). We first filtered to only retain samples with at least 10,000 raw sequencing reads. Then, we specified the following GOTTCHA2 parameters to accommodate our relatively low biomass samples: a cutoff percent of 0.0001 (allowed us to detect very rare taxa), a taxa stringency of 0.5 (allowed for relatively low confidence requirements), an entropy of 0.7 (allowed for more repetitive sequences), per-sample contigs containing at least 100 consecutive bases, and at least 100 reads. Finally, we disabled quality trimming, which is less critical for Nanopore data. We processed the output of the GOTTCHA2 profiling similarly to how NVD output was processed, with several modifications. First, as previously described for NVD-generated data, we performed statistical decontamination using the “combined” method of the “isContaminant()” function in package decontam. Next, we used the “ConQuR” function (with penalized settings) from package ConQuR (v. 2.0) to detect and mitigate batch effects in our data (32). The ConQuR algorithm accounts for within-batch variability in library size and assumes that batch and mean library size are confounded, allowing the use of raw (non-normalized or rarefied) libraries as input data (32). Our samples went through three “batched” processes: RNA extraction, SISPA, and sequencing library preparation. To identify which of these processes imposed the greatest batch effect, we first conducted a permutational analysis of variance (PERMANOVA) using the “adonis2” function in the package vegan. After determining that RNA extraction batch accounted for the largest amount of variation (*R*^2^ = 9.9%, *P* < 0.005), we proceeded with batch correction based on RNA extraction batch. To select an appropriate “reference” batch against which to correct the batch effects of other RNA extraction batches, we tested each RNA extraction batch as a reference batch and calculated the reduction in proportion of variation attributable to batch and the preservation of variation due to covariates (diagnosis, age, fever, cumulative number of symptoms, and clinic visited) after batch correction (Table S2). We did not perform batch correction for NVD-generated virus data because the filtering steps resulted in a data set too sparse to detect batch effects. Following batch correction, we subset the bacterial data set according to the following criteria: (i) retain reads from bacterial species included in a comprehensive list of bacterial pathogens infecting humans (33), (ii) taxon must have at least 10 reads in a sample, and (iii) taxon must have at least two contigs in a sample.

To complement our GOTTCHA2 results, we also uploaded raw fastq files to the CZ ID (formerly IDseq) nanopore pipeline (34). CZ ID is a cloud-based, open-source taxon-agnostic bioinformatics pipeline that downsamples to a maximum of 1 million nanopore reads per sample. After running the pipeline, we generated a *Z*-score metric to distinguish reads due to contamination from potential pathogens or commensals in our samples (35). *Z*-score was calculated as the difference between a taxon’s reads in a biological sample and the taxon’s mean number of reads in control samples, divided by the standard deviation of the taxon’s reads in the control samples. We filtered our data set to include taxa with *Z*-scores ≥1 and alignment length (sample reads against reference genomes) of at least 200 nucleotides and to include those represented by at least 10 bases per million in our sample set and at least two reads per sample. We also discarded alignments ≤90% similarity to reference alignments, and those with alignment significance (*E* values) >10^−100^ (the closer an *E* value is to zero, the more significant the alignment).

### Corroborating malaria RDTs with mNGS

We investigated whether our mNGS approach could identify infections diagnosed with RDTs. For malaria (caused by *Plasmodium* spp.), we categorized samples based on their RDT diagnosis (positive, negative, and unknown) or their sequencing results (whether, after applying filtering criteria described above, samples had reads classified as *P*. *vivax, falciparum, knowlesi, malariae,* or *ovale*). While some typhoid RDTs are designed to detect pathogen antigens (indicative of an ongoing infection), many are designed to detect host antibody to the pathogen, which provides information about host exposure history, but not about whether an infection is ongoing. Because our samples were collected as part of normal clinic operations at multiple unrelated clinics, we could not control which tests were used to test sample donors. Therefore, we have no expectation about whether typhoid RDT results should corroborate the results of sequencing, and we do not present results on their concordance.

To investigate discordance between RDT and sequencing results for malaria detection, we mapped all reads from samples across RDT/sequencing concordance categories against six *Plasmodium* reference genomes (three *P. falciparum* assemblies and one assembly each for *P. knowlesi, P. vivax,* and *P. ovale*) using minimap2 (v. 2.30). To validate whether observed alignments represented true *Plasmodium* infections, we selected representative samples from each RDT/sequencing concordance category (*n* = 6 samples) and the sample with the highest alignment rate against any *Plasmodium* genome (*n* = 1 samples) and performed taxonomic classification using Kraken2 (v2.1.2) with a protozoa-based database. Reads classified as *Plasmodium* by Kraken2 were then verified using BLASTn against NCBI’s nt database to determine their true taxonomic origin.

## RESULTS

### Patient population

Samples were collected at the clinics from 486 patients. The population represented in this subset of the samples ranged in age from 2 to 75 (median age = 28) and comprised a sex ratio of approximately 1:2 male:female (155 male, 294 female, and 32 patients for whom sex was not recorded). Of these patients, 78% (377/486) presented to clinics with fever, and 74% (358/486) had, in addition to fever, at least one of the following symptoms: hematuria (bloody urine), difficulty urinating, rash, jaundice, diarrhea, bloody diarrhea, vomiting, joint pain, headache, myalgia (muscle aches), conjunctivitis, malaise, skin hemorrhage, bleeding gums, nosebleed, or edema. At the time of sample collection, 25 patients included in the study tested positive for malaria, and one malaria-positive patient also tested positive for typhoid, the disease caused by the bacteria *S*. Typhi. Twenty-seven patients (including the coinfection case) tested positive for typhoid (one negative), and 282 patients received no diagnosis (or no diagnosis was recorded, Table 1). Patients were not evenly spread across the four clinics included in the study; rather, 15% (72/486), 47% (226/486), 32% (156/486), and 7% (32/486) came from Comprehensive Health Centre Dadin Kowa, Faith Alive Foundation, Famoseg, and Plateau Specialist Hospital clinics, respectively.

### Sequencing performance

**Fig 2:**
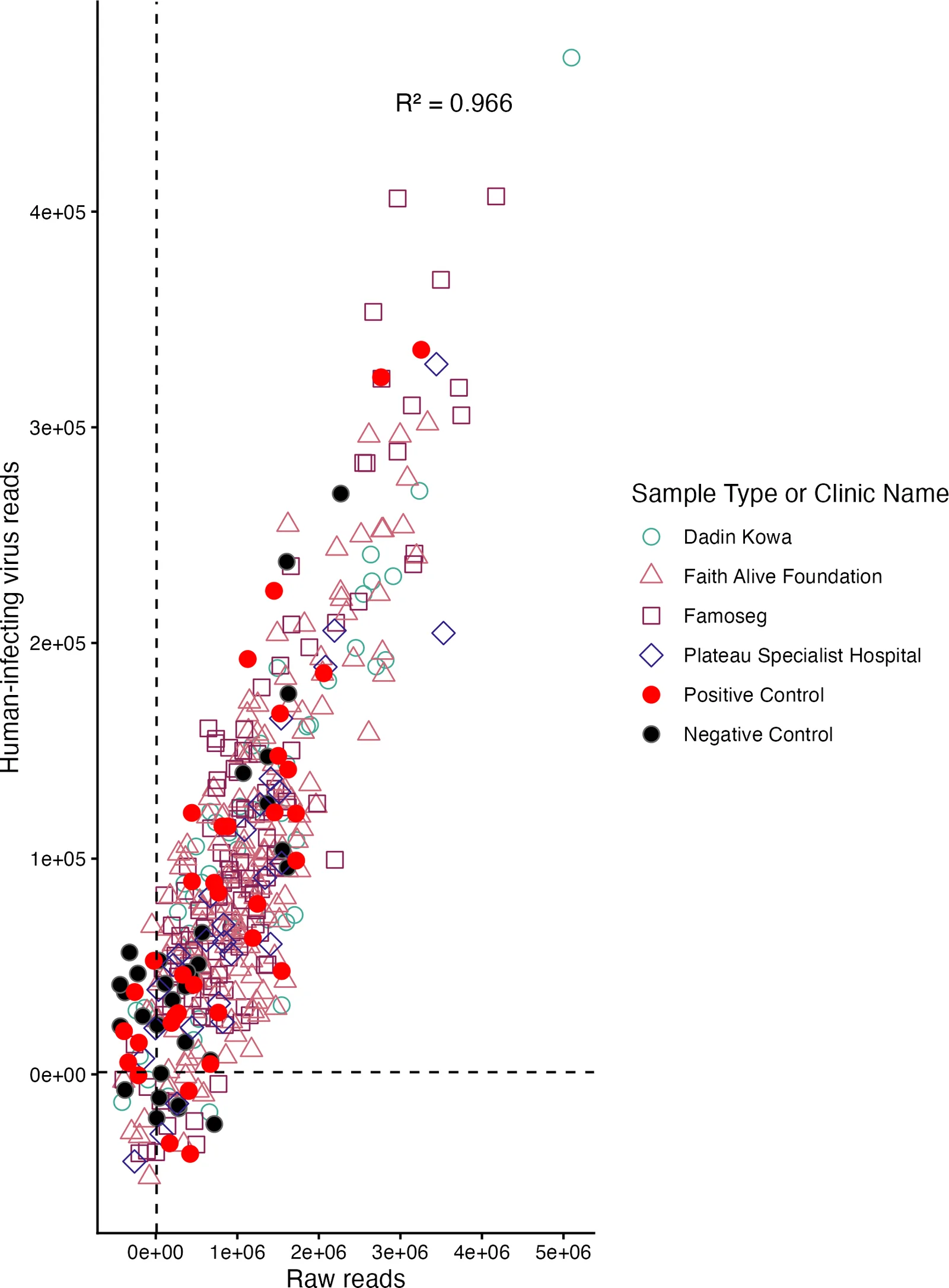
Sequencing depth was strongly associated (*R^2^* = 0.97) with the ability to detect reads from human-infecting viruses. Samples to the right of and above the dashed vertical and horizontal lines contained at least 10,000 and 1,000 raw reads and reads from human-infecting viruses, respectively.

**Fig 3:**
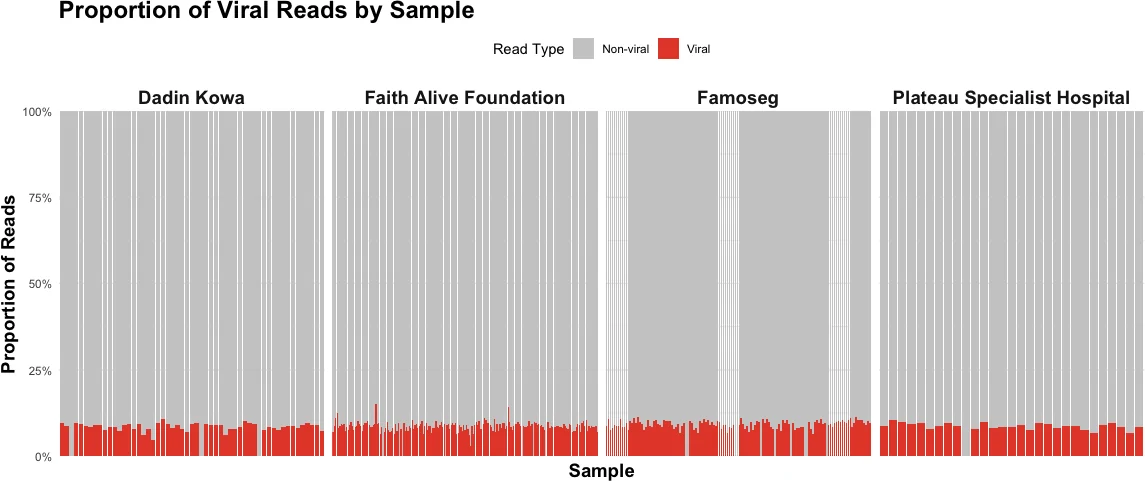
Prior to statistical decontamination, NVD assigned a small proportion (mean = 8.4%) of raw sequencing reads to viruses known to infect humans.

We prepared 13 sequencing libraries and sequenced 417 patient samples, as well as 20 cross-contamination controls, 56 no-template controls, and 19 positive control samples (*n* = 512 samples total). Most libraries predominantly comprised fragments between 800 and 845 bp long (Table S1); however, several libraries (3, 4, 9, and 10 + 11) showed signs of lower quality, indicated by a minority (<50%) of fragments being in this range. Libraries 10 and 11 were combined for both sequencing and fragment analysis. Different subsets of sequencing libraries generated from patient samples were retained for different analyses, depending on analysis-specific filtering criteria. We achieved variable library sizes due to different levels of multiplexing, sequencing runtime, and sample quality (amount of host contamination varied from sample to sample). After discarding libraries with fewer than 10,000 raw reads, we retained 343 sequencing libraries (range = 108,999–5.2 M, mean (SD) = 1.2 M (801,293) reads/library) for NVD analysis. The samples comprising these libraries were representative of the full patient data set (median age = 28, approximate sex ratio 1:2 M:F, 76% febrile, and 72% having fever and one additional symptom). Libraries with greater sequencing depth were more likely to contain reads assigned to human viruses (*R*^2^ = 0.97; Fig. 2). Reads assigned to human viruses were relatively rare, however, comprising 9.0% of all reads in samples (Fig. 3). Cross-contamination rates among sequencing batches were low (mean = 0.15%, median = 0.0005%).

### Viruses

**Fig 4:**
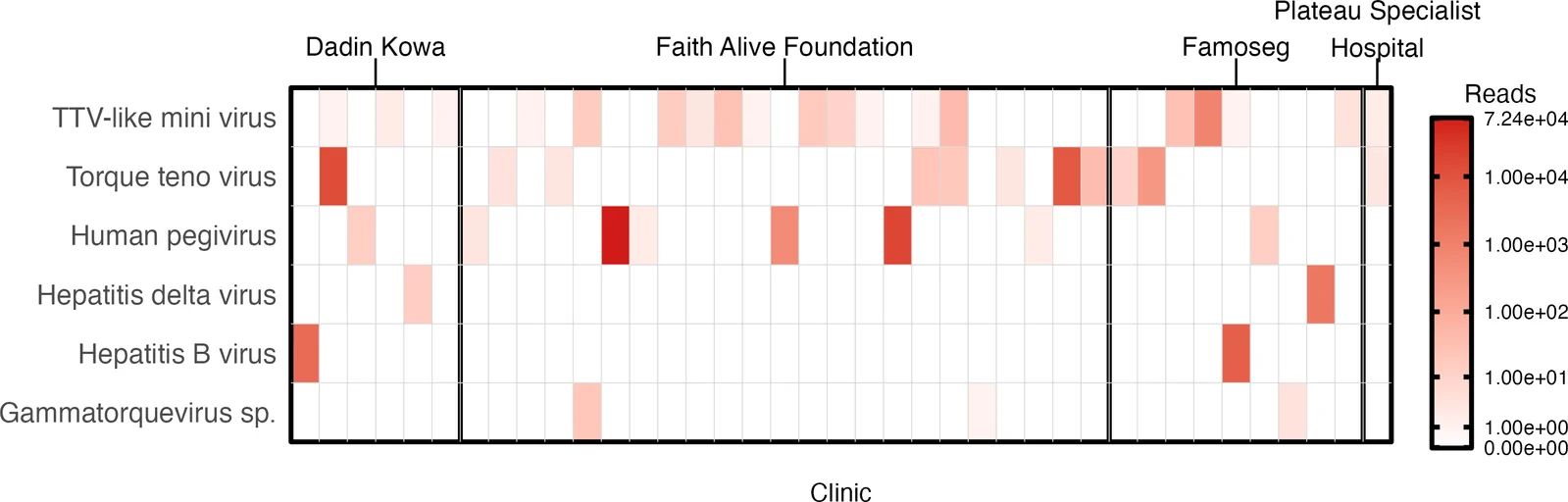
Most human-infecting viruses detected were non-pathogenic anelloviruses. Two patients had reads from hepatitis B virus. Columns representing individual patients are grouped by clinic. Box color saturation indicates the number of reads classified as each virus (rows), with more saturated colors corresponding to more sequencing reads for a virus × patient combination. Only patients with human-infecting virus reads are shown.

**Fig 5:**
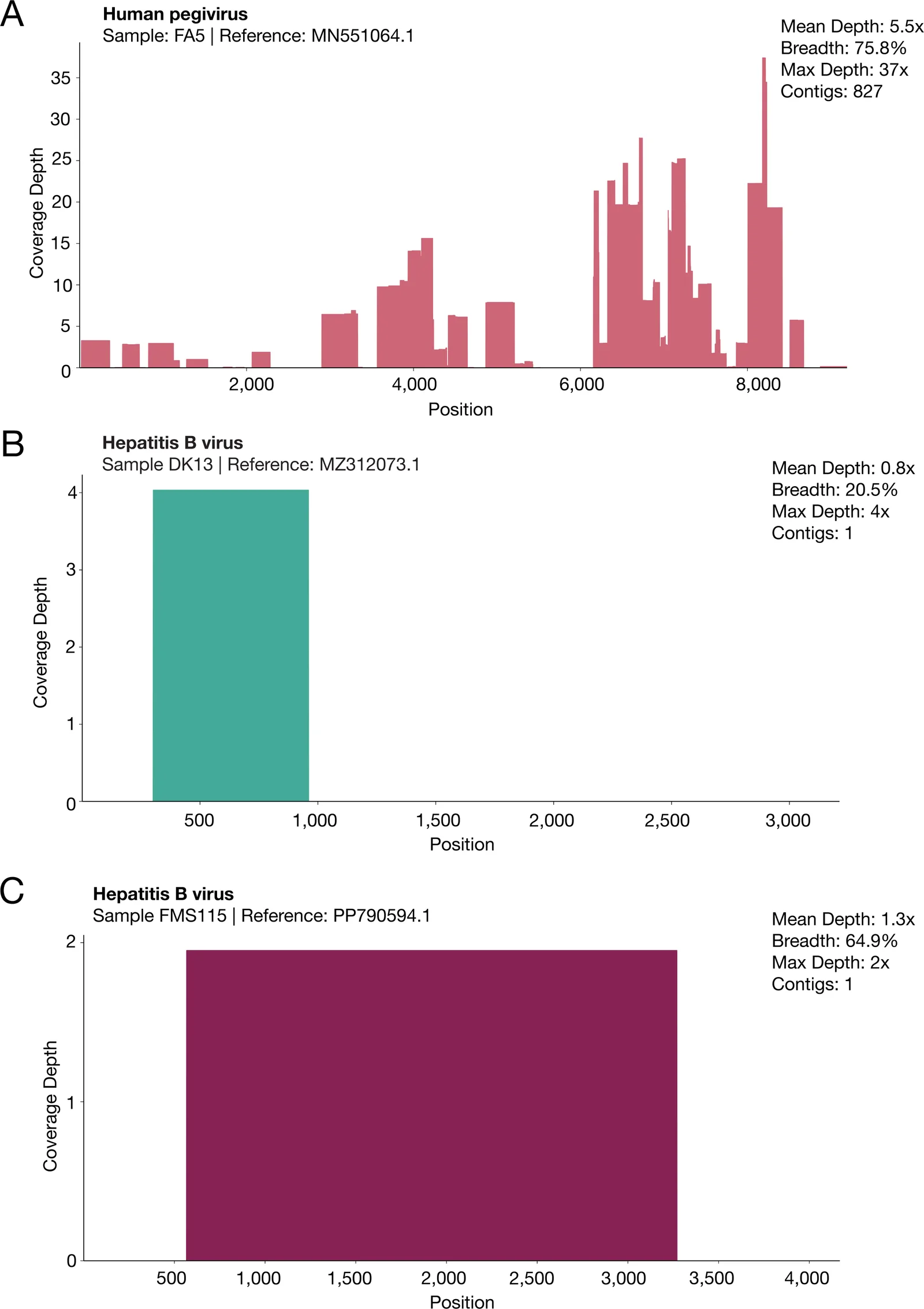
Representative coverage plots illustrating genomic coverage of (**A**) human pegivirus reads recovered from sample FA5, (**B**) hepatitis B virus reads recovered from sample DK13, and (**C**) hepatitis B virus reads recovered from sample FMS115. Reference genome accessions against which reads were mapped are listed in plot subtitles.

After filtering reads according to the criteria described previously, we retained six viral taxa in 39/343 samples (Fig. 4), representing only 11% of the patient samples with usable sequencing data. We identified one known vertebrate virus, hepatitis B virus (HBV; *n* = 2), as well as five human viruses not currently believed to cause disease: torque teno virus (TTV; *n* = 11), TTV-like mini virus (*n* = 19), human pegivirus (*Pegivirus hominis,* HPgV; *n* = 8), hepatitis delta virus (*n* = 2), and species in the *Gammotorquevirus* genus (*n* = 3). The most frequently detected virus was TTV-like mini virus in 5.5% (19/343) of samples that produced usable sequencing data. The virus with the highest read count in one sample was HPgV in sample FA5, where there were 72,425 reads classified as being from HPgV. The HPgV reads recovered from this patient’s sample covered approximately 76% (average depth 37×) of an HPgV genome isolate identified in Nigeria in 2018 (Fig. 5A). Prevalence of HPgV in our sample set was low (~2.3%, 8/343). We detected sequences from HBV in samples from two patients (Fig. 4). In one patient, DK13, the 3,235 reads classified as HBV aligned with >98% similarity (and with equal coverage) to HBV isolates from other patients from Nigeria and Cameroon (36–38) (Fig. 5B). In the second patient with HBV reads, FMS115, the 5,552 reads mapped with >98% similarity to an HBV sub-genotype A clone generated by a South African research group (GenBank accession PP790594.1; Fig. 5C). Both patients with reads classified as HBV were febrile and reported having headaches, and patient DK13 additionally reported joint pain and myalgia. Data on malaria and typhoid positivity status were unavailable for both patients.

### Taxon-agnostic analysis results

We used different filtering criteria to parse our GOTTCHA2-derived results and our CZ ID-derived results, following general recommendations from other groups who have performed mNGS studies for our CZ ID filtering. Prior to applying the “potential pathogen” filter, our CZ ID data set contained 359 samples (Table 1). After filtering to retain only “potential pathogen”-flagged taxa, our data set contained 315 samples and 83 taxa, of which 4 were viral, 73 were bacterial, and 6 were eukaryotic. Two samples (FMS159 and F17) that were successfully analyzed by NVD repeatedly failed to pass through the CZID analysis pipeline and were discarded. For parsing GOTTCHA2 data, we used filtering and decontamination criteria described in the methods, retaining only bacterial taxa with the potential to infect humans. This resulted in a data set of 52 samples and 53 bacterial taxa (Table S3), which we further investigated for potential bacterial infections.

Bacterial taxa remaining in this data set were represented in each sample by at least 2 contigs and 10 reads and were classified as species included in a published list of bacterial pathogens infectious to humans (33). Read depth and breadth of human-infectious bacteria were relatively low; the maximum number of reads assigned to any single taxon in a sample was 20, and median coverage breadth (positions of a reference genome recovered in our sequencing data) was 2,500 bases. The most frequently detected bacterial species in this reduced data set was *Enterobacter kobei*, which we observed in 12/343 (3.5 %) samples. This taxon also had the greatest coverage in our data set; in one sample (FMS116), *Enterobacter kobei* reads covered 14,665 positions (0.3% of the 4.8 Mb genome) of the two reference genomes to which our reads BLASTed.

### Malaria RDT concordance

Decontamination filtering of our GOTTCHA2-derived data classified all *Plasmodium* spp. reads as contamination; all references to *Plasmodium* parasites detected with sequencing data going forward pertain to alignments that passed CZ ID filtering steps (described in methods). Among samples from patients with recorded RDT diagnoses for malaria (*n* = 21 positive, 119 negative), we found poor concordance between RDT and sequencing results (Table S3). Of the 21 patients who tested malaria-positive via RDT, only one (FA10) also had reads that passed our filters and mapped to *P. falciparum* in CZ ID. Among the 119 patients who tested negative for malaria via RDT, we detected filter-passing reads from *P. falciparum* in five samples.

Alignment rates to *P. falciparum* (3D7) references were uniformly low across all sample categories, ranging from 0.01% to 1.22% with no clear distinction between RDT-positive and RDT-negative samples. Samples showed substantially higher alignment rates to *P. knowlesi* (0.1%–42.8%) and *P. vivax* (0.09%–19.4%) references compared to *P. falciparum*, with this pattern persisting even in RDT-negative control samples.

BLAST validation of Kraken2-classified *Plasmodium* reads from representative samples (one per RDT/sequencing concordance category) showed that these sequences were predominantly human (84%) or other primate sequences (9%–16% *Macaca*), with zero reads confirmed as authentic *Plasmodium* species. Investigation of FAL2, an RDT-negative sample showing the highest alignment rate to *P. knowlesi* (42.8%), revealed that reads mapping to the apicoplast genome were bacterial sequences (*Achromobacter* spp.), confirming that high alignment rates resulted from contamination rather than true *Plasmodium* infection. This pattern was consistent across all tested samples regardless of RDT or sequencing status, indicating that the observed minimap2 alignments to *Plasmodium* genomes represent non-specific mapping of human, primate, and bacterial reads rather than true malaria parasite signal.

## DISCUSSION

We used metagenomic sequencing of RNA from human serum samples to detect the potential causes of febrile illness. Definitive diagnosis of the infectious etiology of a patient’s symptoms is beyond the confirmatory abilities of the data generated with mNGS techniques. Under ideal circumstances, however, such data could be used to narrow the range of or even pinpoint an etiological culprit which might then be confirmed with culture-, antigen-, microscopy-based, or targeted molecular diagnostic testing. The data included in the present study represent a culmination of several suboptimal, but not uncommon, circumstances that may have influenced our ability to detect potential microbial sources of febrile illness. These circumstances, including uncertainty and complications surrounding sample storage conditions during export, as well as host and laboratory contamination, may have constrained our ability to isolate, sequence, and identify RNA from every potentially pathogenic microbe in the data set. Substantial variation in sequencing depth has been reported in previous mNGS studies of clinical samples, ranging from thresholds as low as 1,000 reads/sample to at least 2 million reads/sample. Our average sequencing depth of 1.2 million reads per sample falls within this range and is consistent with other recent studies in which negative results were common (14, 15).

Sample quality in our study may have been compromised prior to RNA extraction. During export and transport from Nigeria to the United States, the courier tasked with shipping samples experienced delays, during which time the cold chain of sample storage may have been disrupted. This suspicion is impossible to determine unequivocally, however, the sparse and relatively fragmented microbial nucleic acid content extracted from our samples suggests that some sample degradation may have occurred during transit. While an RNA/DNA-stabilizing agent could have lessened the likelihood and extent of sample degradation, had we detected arbovirus RNA in samples, our next step would have been to attempt to isolate, grow, characterize, and sequence the genomes of these viruses. Using a stabilizing agent or fixative would have precluded this ability, illustrating the tradeoff between fixing and preserving nucleic acids and maximizing the ability to use biological samples for multiple purposes. In light of the challenges posed by possible degradation of samples, we suggest that a short-read platform such as Illumina, designed to sequence PCR-amplified shorter fragments at high depths, may have yielded more usable data than what we generated using a long-read platform. This approach too would have incurred tradeoffs, however, such as possible amplification and sequencing biases.

Independent of issues related to sample transit and storage, our samples overwhelmingly yielded host (human) reads. This issue is common among metagenomic pathogen surveys from human tissues (35, 39), especially from low biomass sample materials such as serum. The percent of reads in our study classified as being from viruses potentially pathogenic to humans (8.4%) fell within the range reported by Grundy et al. (35). While our analysis using NVD essentially performs *in silico* host depletion by white-listing for reads that come from human-infecting virus families, we did not perform any host-depletion steps during wet lab sample preparation. Other investigators have successfully dealt with host read contamination by performing host depletion or viral capture and enrichment processes on patient samples (40); however, including such a step is not without tradeoffs. Every additional procedure to which samples are subjected increases the potential for “kit-ome” contamination and imposes additional requirements in labor, time, expense, and expertise, which may make projects less feasible in resource-limited settings (41). Metagenomic sequencing libraries of nucleic acid from low-biomass samples, such as serum, are extremely susceptible to non-host contamination as well (41). To counter this, we used negative controls at every step of laboratory work to control for contamination of reagents and workspaces. Unfortunately, we could not control for potential contamination at the clinic level. Our GOTTCHA2 analysis PERMANOVA estimate indicated that there was a strong effect of clinic on the bacterial reads detected in patient sera. It is possible that patients from different parts of Jos, who presumably visited the clinics closest to their dwellings, do have relatively distinct microbial communities, but it is much more likely that the clinics themselves varied in the suite of contaminants to which samples collected at those clinics were exposed.

Despite methodological challenges, we did detect several viral taxa that, based on other mNGS surveys of human sera, we were unsurprised to find in our samples. For example, others have frequently detected pegiviruses in metagenomic surveys of human serum (13–15, 39). We detected HPgV in 8/343 (~2.3%) samples with usable sequence data. HPgV is a common, apparently nonpathogenic virus that can cause long-lasting high-titer viremia but links to disease are unsubstantiated (42). While we do not interpret detections of HPgV to be informative from the standpoint of identifying an etiological source of disease, we do consider its detection to be an indication that our methodological approaches were effective when sample quality was adequate, and nucleic acid from pathogens was present in sufficient quantities to detect.

Our conclusions regarding the possible presence of bacterial pathogens in our samples are less certain. Even after employing the same laboratory control scheme and even more stringent *in silico* filtering and decontamination methods as were used for data on viral reads, the bacterial reads we most often detected in our samples were infrequent human pathogens. The most commonly detected bacterium, *Enterobacter kobei,* was seen in 12 samples (3.4% of samples with usable sequencing data). In patient FMS116, *E. kobei* was represented by two contigs that BLASTed to two different reference genomes. Neither of these genomes was Nigerian in origin, and only one was isolated from a human infection (the other was isolated from an environmental source). Additionally, most published reports of *E. kobei* describe nosocomial infections, but our study did not collect the detailed health histories needed to assess this likelihood (43, 44). Our bacterial reads are difficult to interpret definitively, as uncommon bacterial infections are rarely diagnosed, and even more rarely sequenced and archived. While bacterial infections, especially *Salmonella*, are a leading cause of febrile illness in Sub-Saharan Africa (45), we emphasize the importance of interpreting detections of human-infecting bacteria cautiously. On the flip side, given the sample-related challenges described previously, it is difficult to know whether “negative” sequencing data (samples in which no fever-causing pathogen RNA is detected) truly represent the absence of pathogens or simply the failure to detect them.

We leveraged the heterogeneity within our patient data with respect to RDT diagnosis for malaria by conducting a concordance analysis. Specifically, we investigated whether our sequencing approach detected sequences from human-infecting *Plasmodium* species in samples from patients who tested positive for infections with these pathogens via RDT. In all cases, we concluded that our sequencing did not produce sequences from *Plasmodium* spp.; however, had we treated initial CZ ID or GOTTCHA2 results as final, bypassing additional blastn checks and stringent contamination filtering and batch effect mediation, respectively, we would have considered *Plasmodium* spp. infections to be prevalent in our sample set. We stress that failure to detect sequences from these infectious agents in samples from patients with known positive infection status does not nullify the validity of RDT results. Rather, this outcome is indicative of biological characteristics of the parasite and limitations of our study design. Our investigation of RDT vs sequencing concordance was opportunistic and highlights the importance of rigorous bioinformatic filtering steps before accepting that an infectious agent is truly present in a sample. Finally, while *Plasmodium* detection and an evaluation of the effectiveness of *Plasmodium* RDTs were not our study goals, if they had been, a *Plasmodium*-based qPCR would provide even more information on the sensitivity of the *Plasmodium* RDTs used in this study. As intracellular parasites, *Plasmodium* spp. are unlikely to be present in the cell-free sample material (serum) used in our study.

Researchers have successfully used mNGS to identify etiological culprits across different patient populations, sample types, and hospital settings (inpatient versus outpatient). For example, using cerebrospinal fluid of pediatric patients with idiopathic meningitis in Bangladesh, Saha and colleagues (46) identified previously undetected neuroinvasive Chikungunya virus infections. In Edo State, Nigeria, a rash of patients with unexplained febrile illness who presented to a clinic in the Lassa-endemic region were found to be infected with yellow fever virus using mNGS to a comparable sequencing depth (1.6 M reads/sample) to that used in our study (1.2 M reads/sample) (47). Unlike these examples, in which mNGS was deployed in response to unexplained public health phenomena, our study is more representative of a routine surveillance scenario where the data could then be used to identify which pathogens to prioritize for diagnostic capacity and development of molecular assays. Our study was conducted on a cross-section of the patient population visiting four clinics in the capital city of Plateau State, Nigeria, in 2023. Rather than responding to a surge of unexplained febrile illnesses in a demographic or temporal focus, our samples were collected opportunistically as patients visited clinics of their own volition to seek care. Therefore, despite the geographical proximity from which they originated, the samples used in our study are heterogeneous in several potentially consequential dimensions. Age, sex, and prior infection status all varied in our patient cohort. Our samples came from ambulatory outpatients who were able to get themselves (or be taken by their caretakers) to clinics, introducing another potential source of sample heterogeneity: the elapsed time between symptom onset, clinic visitation, and sample collection. Without standardization of the timing of sample collection, fever-associated viremia or bacteremia could have cleared prior to sample collection. In a different study of febrile patients in southern Nigeria, mNGS only detected dengue virus (DENV) reads in ~58% of serologically positive DENV samples tested, indicating that even confirmed infections are often not detected with mNGS (48).

Our findings lend insight to several important considerations for mNGS investigations and researchers. During our investigation, we were several times reminded that the exquisite sensitivity of mNGS can be a double-edged blade. On one hand, we show that despite the microbial sparsity and low biomass of the sample type we used (serum) and the minimal processing (intended to make our approach easy to deploy in low-resource settings) we applied to our samples, we were able to detect the presence of viruses known to infect humans. On the other hand, we also detected insidious lab-based contamination at very low (below qPCR detectability) levels from pathogenic viruses with endemicity to our study area. Specifically, we detected RNA from West Nile virus (WNV) and DENV in several samples. These viruses are known to circulate in Nigeria and are also among the viruses studied by researchers in the same physical space where we conducted the lab work for this study. Even after statistical contaminant removal and nucleotide BLAST searches on the sequences classified as WNV and DENV, ambiguity remained surrounding their origins (was their presence due to lab contamination or were they truly present in patient samples?). To answer this question with greater confidence, we used a tiled PCR (PrimalSeq) approach, following the methods of Quick et al. (49) and Vogels et al. (50), to attempt to recover and sequence the entire viral genomes of WNV and DENV from putative-positive samples (supplemental methods). This approach allowed us to definitively rule out the possibility that the sequences were true positives (supplemental results).

Despite diligent protocols for cleaning shared lab spaces, the use of negative controls at every step of the laboratory work, and employing post-sequencing bioinformatic methods for contamination removal, we found that trace amounts of fragmented RNA can still contaminate mNGS pipelines, potentially leading to false positives. Additionally, the labor and time required to rule out such spurious positives is considerable, suggesting that ideal implementations of mNGS approaches will include dedicated facilities, which may often be unrealistic. Furthermore, the preponderance of false leads and contaminants we detected in our study is likely an artifact of using patients’ sera, a low-biomass material, as our sample source, and due to the fact that we did not attempt to match sample type to patient symptomology. We collected serum, regardless of what symptoms patients presented in addition to fever; had we been able to collect other sample types such as saliva, feces, or whole blood, we likely would have detected many more pathogens, and perhaps could have detected concordance between infection etiology and patient symptomology. Given these challenges, it is perhaps unsurprising that the sequencing we performed primarily detected the presence of nonpathogenic commensal viruses (after controlling for potential contaminants). In fact, we suggest that the sensitivity of mNGS to timing of sample collection, sample type, susceptibility to contamination at each stage of the process, and required expertise for method implementation may make it most informative at far ends of the use case spectrum. For instance, mNGS may be valuable as a surveillance tool for samples collected in aggregate settings such as air and wastewater or a highly targeted tool for individual patient samples during acute and well-monitored unexplained illnesses; however, the challenges we outline may hamper the technique’s utility for routine clinic-level surveillance efforts.

### Conclusion

Our goal in this study was to use mNGS to detect and identify possible causative agents of febrile illness among patients who presented to four clinics in Jos, Plateau State, Nigeria. We were able to detect common human-infecting viruses unlikely to cause fever, but only in a small subset of the samples we considered. Potential bacterial sources of fever and other symptoms were more commonly detected; however, the coverage of bacterial genomes was low, and symptoms due to bacteremia would need to be confirmed with follow-up cultures or qPCR. We suggest that in aggregate, public health monitoring settings, long-term surveillance data generated with mNGS could provide valuable baseline estimates of expected microbial communities, allowing researchers and public health workers to flag potential pathogens of concern. We also recognize the utility of mNGS technology as a method for narrowing the range of potential etiologies of disease or pathology outbreaks in well-defined patient populations. Without capacity to provide a secondary, definitive diagnosis, the time, expertise, and resources required to carry out mNGS investigations may make them unsuitable as one-off additions to the clinician’s diagnostic toolkit, especially in resource-limited settings or when other patient data are sparse.

## Data Availability

The sequencing data generated in this study have been deposited in the NCBI Sequence Read Archive (SRA) under BioProject ID PRJNA1417234.

## SUPPLEMENTAL MATERIAL

Data S1 msphere.00157-26-s0001.xlsx Synthetic RNA spike-in controls.

Supplemental material msphere.00157-26-s0002.docx Supplemental methods and results, Tables S1 to S4, and Fig. S1 and S2.

ASM does not own the copyrights to Supplemental Material that may be linked to, or accessed through, an article. The authors have granted ASM a non-exclusive, world-wide license to publish the Supplemental Material files. Please contact the corresponding author directly for reuse.

## References

[1] Barathan M. 2024. From fever to action: diagnosis, treatment, and prevention of acute undifferentiated febrile illnesses. Pathog Dis 82:ftae006. doi:10.1093/femspd/ftae006 PMC11067964 38614961

[2] Prasad N, Murdoch DR, Reyburn H, Crump JA. 2015. Etiology of severe febrile illness in low- and middle-income countries: a systematic review. PLoS One 10:e0127962. doi:10.1371/journal.pone.0127962 PMC4488327 26126200

[3] Eigege A, Richards FO, Blaney DD, Miri ES, Gontor I, Ogah G, Umaru J, Jinadu MY, Mathai W, Amadiegwu S, Hopkins DR. 2003. Rapid assessment for lymphatic filariasis in central Nigeria: a comparison of the immunochromatographic card test and hydrocele rates in an area of high endemicity. Am J Trop Med Hyg 68:643–646. doi:10.4269/ajtmh.2003.68.643 12887020

[4] Mfuh KO, Abanda NN, Titanji BK. 2023. Strengthening diagnostic capacity in Africa as a key pillar of public health and pandemic preparedness. PLOS Glob Public Health 3:e0001998. doi:10.1371/journal.pgph.0001998 PMC10263319 37310963

[5] Romano ER, Sleeman K, Hall-Eidson P, Zeh C, Bhairavabhotla R, Zhang G, Adhikari A, Alemnji G, Cardo YR, Pinheiro A, et al.. 2022. Contribution of PEPFAR-supported HIV and TB molecular diagnostic networks to COVID-19 testing preparedness in 16 countries. Emerg Infect Dis 28:S59–S68. doi:10.3201/eid2813.220789 PMC9745242 36502414

[6] Mayor A, Bassat Q. 2019. “Resistance” to diagnostics: a serious biological challenge for malaria control and elimination. EBioMedicine 50:9–10. doi:10.1016/j.ebiom.2019.11.041 PMC6921289 31812497

[7] Wainaina M, Vey da Silva DA, Dohoo I, Mayer-Scholl A, Roesel K, Hofreuter D, Roesler U, Lindahl J, Bett B, Al Dahouk S. 2022. A systematic review and meta-analysis of the aetiological agents of non-malarial febrile illnesses in Africa. PLoS Negl Trop Dis 16:e0010144. doi:10.1371/journal.pntd.0010144 PMC8812962 35073309

[8] Gauthier NPG, Chorlton SD, Krajden M, Manges AR. 2023. Agnostic sequencing for detection of viral pathogens. Clin Microbiol Rev 36:e0011922. doi:10.1128/cmr.00119-22 PMC10035330 36847515

[9] Mwamba D, Akilimali P, Mboussou F, Kabasubabo F, Angendu K, Ngandu C, Fotsing R, Ancia A, Kamba RS. 2026. Bundibugyo virus disease outbreak in Ituri, Democratic Republic of the Congo. Lancet 407:2367–2369. doi:10.1016/S0140-6736(26)01072-X 42214396

[10] Dondorp AM, Nosten F, Yi P, Das D, Phyo AP, Tarning J, Lwin KM, Ariey F, Hanpithakpong W, Lee SJ, Ringwald P, Silamut K, Imwong M, Chotivanich K, Lim P, Herdman T, An SS, Yeung S, Singhasivanon P, Day NPJ, Lindegardh N, Socheat D, White NJ. 2009. Artemisinin resistance in *Plasmodium falciparum* malaria. N Engl J Med 361:455–467. doi:10.1056/NEJMoa0808859 PMC3495232 19641202

[11] Uwimana A, Legrand E, Stokes BH, Ndikumana J-LM, Warsame M, Umulisa N, Ngamije D, Munyaneza T, Mazarati J-B, Munguti K, Campagne P, Criscuolo A, Ariey F, Murindahabi M, Ringwald P, Fidock DA, Mbituyumuremyi A, Menard D. 2020. Emergence and clonal expansion of *in vitro* artemisinin-resistant *Plasmodium falciparum* kelch13 R561H mutant parasites in Rwanda. Nat Med 26:1602–1608. doi:10.1038/s41591-020-1005-2 PMC7541349 32747827

[12] Oladosu OO, Oyibo WA. 2013. Overdiagnosis and overtreatment of malaria in children that presented with fever in Lagos, Nigeria. ISRN Infect Dis 2013:1–6. doi:10.5402/2013/914675

[13] Camprubí-Ferrer D, Tomazatos A, Balerdi-Sarasola L, Cobuccio LG, Van Den Broucke S, Horváth B, Van Esbroeck M, Martinez MJ, Gandasegui J, Subirà C, Saloni M, Genton B, Bottieau E, Cadar D, Muñoz J. 2024. Assessing viral metagenomics for the diagnosis of acute undifferentiated fever in returned travellers: a multicenter cohort study. J Travel Med 31:taae029. doi:10.1093/jtm/taae029 38381609

[14] Levine ZC, Sene A, Mkandawire W, Deme AB, Ndiaye T, Sy M, Gaye A, Diedhiou Y, Mbaye AM, Ndiaye IM, Gomis J, Ndiop M, Sene D, Faye Paye M, MacInnis BL, Schaffner SF, Park DJ, Badiane AS, Colubri A, Ndiaye M, Sy N, Sabeti PC, Ndiaye D, Siddle KJ. 2024. Investigating the etiologies of non-malarial febrile illness in Senegal using metagenomic sequencing. Nat Commun 15:747. doi:10.1038/s41467-024-44800-7 PMC10810818 38272885

[15] Oguzie JU, Petros BA, Oluniyi PE, Mehta SB, Eromon PE, Nair P, Adewale-Fasoro O, Ifoga PD, Odia I, Pastusiak A, et al.. 2023. Metagenomic surveillance uncovers diverse and novel viral taxa in febrile patients from Nigeria. Nat Commun 14:4693. doi:10.1038/s41467-023-40247-4 PMC10403498 37542071

[16] McCall C, Wu H, Miyani B, Xagoraraki I. 2020. Identification of multiple potential viral diseases in a large urban center using wastewater surveillance. Water Res 184:116160. doi:10.1016/j.watres.2020.116160 PMC7342010 32738707

[17] Minor NR, Ramuta MD, Stauss MR, Harwood OE, Brakefield SF, Alberts A, Vuyk WC, Bobholz MJ, Rosinski JR, Wolf S, Lund M, Mussa M, Beversdorf LJ, Aliota MT, O’Connor SL, O’Connor DH. 2023. Metagenomic sequencing detects human respiratory and enteric viruses in air samples collected from congregate settings. Sci Rep 13:21398. doi:10.1038/s41598-023-48352-6 PMC10696062 38049453

[18] Weetman D, Kamgang B, Badolo A, Moyes CL, Shearer FM, Coulibaly M, Pinto J, Lambrechts L, McCall PJ. 2018. *Aedes* mosquitoes and *Aedes*-borne arboviruses in Africa: current and future threats. Int J Environ Res Public Health 15:220. doi:10.3390/ijerph15020220 PMC5858289 29382107

[19] Cordey S, Laubscher F, Hartley M-A, Junier T, Keitel K, Docquier M, Guex N, Iseli C, Vieille G, Le Mercier P, Gleizes A, Samaka J, Mlaganile T, Kagoro F, Masimba J, Said Z, Temba H, Elbanna GH, Tapparel C, Zanella M-C, Xenarios I, Fellay J, D’Acremont V, Kaiser L. 2021. Blood virosphere in febrile Tanzanian children. Emerg Microbes Infect 10:982–993. doi:10.1080/22221751.2021.1925161 PMC8171259 33929935

[20] Duffy MR, Chen T-H, Hancock WT, Powers AM, Kool JL, Lanciotti RS, Pretrick M, Marfel M, Holzbauer S, Dubray C, Guillaumot L, Griggs A, Bel M, Lambert AJ, Laven J, Kosoy O, Panella A, Biggerstaff BJ, Fischer M, Hayes EB. 2009. Zika virus outbreak on Yap Island, Federated States of Micronesia. N Engl J Med 360:2536–2543. doi:10.1056/NEJMoa0805715 19516034

[21] Mostashari F, Bunning ML, Kitsutani PT, Singer DA, Nash D, Cooper MJ, Katz N, Liljebjelke KA, Biggerstaff BJ, Fine AD, Layton MC, Mullin SM, Johnson AJ, Martin DA, Hayes EB, Campbell GL. 2001. Epidemic West Nile encephalitis, New York, 1999: results of a household-based seroepidemiological survey. Lancet 358:261–264. doi:10.1016/S0140-6736(01)05480-0 11498211

[22] De Santis O, Bouscaren N, Flahault A. 2023. Asymptomatic dengue infection rate: a systematic literature review. Heliyon 9:e20069. doi:10.1016/j.heliyon.2023.e20069 PMC10559824 37809992

[23] Ukaegbu CO, Nnachi AU, Mawak JD, Igwe CC. 2014. Incidence of concurrent malaria and typhoid fever infections in febrile patients in Jos, Plateau State Nigeria. Int J Sci Technol Res 3:157–161.

[24] Chomczynski P, Sacchi N. 1987. Single-step method of RNA isolation by acid guanidinium thiocyanate-phenol-chloroform extraction. Anal Biochem 162:156–159. doi:10.1006/abio.1987.9999 2440339

[25] Kafetzopoulou LE, Efthymiadis K, Lewandowski K, Crook A, Carter D, Osborne J, Aarons E, Hewson R, Hiscox JA, Carroll MW, Vipond R, Pullan ST. 2018. Assessment of metagenomic Nanopore and Illumina sequencing for recovering whole genome sequences of chikungunya and dengue viruses directly from clinical samples. Euro Surveill 23:1800228. doi:10.2807/1560-7917.ES.2018.23.50.1800228 PMC6299504 30563591

[26] Lewandowski K, Xu Y, Pullan ST, Lumley SF, Foster D, Sanderson N, Vaughan A, Morgan M, Bright N, Kavanagh J, Vipond R, Carroll M, Marriott AC, Gooch KE, Andersson M, Jeffery K, Peto TEA, Crook DW, Walker AS, Matthews PC. 2019. Metagenomic nanopore sequencing of influenza virus direct from clinical respiratory samples. J Clin Microbiol 58:e00963-19. doi:10.1128/JCM.00963-19 PMC6935926 31666364

[27] Lagerborg KA, Normandin E, Bauer MR, Adams G, Figueroa K, Loreth C, Gladden-Young A, Shaw BM, Pearlman LR, Berenzy D, Dewey HB, Kales S, Dobbins ST, Shenoy ES, Hooper D, Pierce VM, Zachary KC, Park DJ, MacInnis BL, Tewhey R, Lemieux JE, Sabeti PC, Reilly SK, Siddle KJ. 2022. Synthetic DNA spike-ins (SDSIs) enable sample tracking and detection of inter-sample contamination in SARS-CoV-2 sequencing workflows. Nat Microbiol 7:108–119. doi:10.1038/s41564-021-01019-2 PMC8923058 34907347

[28] Katz KS, Shutov O, Lapoint R, Kimelman M, Brister JR, O’Sullivan C. 2021. STAT: a fast, scalable, MinHash-based *k*-mer tool to assess sequence read archive next-generation sequence submissions. Genome Biol 22:270. doi:10.1186/s13059-021-02490-0 PMC8450716 34544477

[29] McMurdie PJ, Holmes SP. 2013. Phyloseq: an R package for reproducible interactive analysis and graphics of microbiome census data. PLoS One 8:e61217. doi:10.1371/journal.pone.0061217 PMC3632530 23630581

[30] Davis NM, Proctor DM, Holmes SP, Relman DA, Callahan BJ. 2018. Simple statistical identification and removal of contaminant sequences in marker-gene and metagenomics data. Microbiome 6:226. doi:10.1186/s40168-018-0605-2 PMC6298009 30558668

[31] Freitas TAK, Li P-E, Scholz MB, Chain PSG. 2015. Accurate read-based metagenome characterization using a hierarchical suite of unique signatures. Nucleic Acids Res 43:e69. doi:10.1093/nar/gkv180 PMC4446416 25765641

[32] Ling W, Lu J, Zhao N, Lulla A, Plantinga AM, Fu W, Zhang A, Liu H, Song H, Li Z, Chen J, Randolph TW, Koay WLA, White JR, Launer LJ, Fodor AA, Meyer KA, Wu MC. 2022. Batch effects removal for microbiome data via conditional quantile regression. Nat Commun 13:5418. doi:10.1038/s41467-022-33071-9 PMC9477887 36109499

[33] Bartlett A, Padfield D, Lear L, Bendall R, Vos M. 2022. A comprehensive list of bacterial pathogens infecting humans. Microbiology (Reading) 168. doi:10.1099/mic.0.001269 36748702

[34] Simmonds SE, Ly L, Beaulaurier J, Lim R, Morse T, Thakku SG, Rosario K, Perez JC, Puschnik A, Mwakibete L, Hickey S, Tato CM, Team CI, Kalantar K. 2024. CZ ID: a cloud-based, no-code platform enabling advanced long read metagenomic analysis. bioRxiv. doi:10.1101/2024.02.29.579666

[35] Grundy BS, Parikh H, Jacob S, Banura P, Moore CC, Liu J, Houpt ER. 2023. Pathogen detection using metagenomic next-generation sequencing of plasma samples from patients with sepsis in Uganda. Microbiol Spectr 11:e04312-22. doi:10.1128/spectrum.04312-22 PMC9927450 36625651

[36] Zafrullah M, Vazquez C, Mixson-Hayden T, Purdy MA. 2021. *In vitro* characterization of six hepatitis B virus genotypes from clinical isolates using transfecting linear HBV genomes. J Gen Virol 102:001675. doi:10.1099/jgv.0.001675 34723786

[37] Forbi JC, Vaughan G, Purdy MA, Campo DS, Xia G, Ganova-Raeva LM, Ramachandran S, Thai H, Khudyakov YE. 2010. Epidemic history and evolutionary dynamics of hepatitis B virus infection in two remote communities in rural Nigeria. PLoS One 5:e11615. doi:10.1371/journal.pone.0011615 PMC2906510 20657838

[38] Shimakawa Y, Veillon P, Birguel J, Pivert A, Sauvage V, Guillou-Guillemette HL, Roger S, Njouom R, Ducancelle A, Amta P, Huraux JM, Adoukara J-P, Lunel-Fabiani F. 2022. Residual risk of mother-to-child transmission of hepatitis B virus infection despite timely birth-dose vaccination in Cameroon (ANRS 12303): a single-centre, longitudinal observational study. Lancet Glob Health 10:e521–e529. doi:10.1016/S2214-109X(22)00026-2 35183302

[39] Mziray SR, Githinji G, de Laurent ZR, Mbelele PM, Mohammed KS, Wadugu BD, Grundy BS, Heysell SK, Mpagama SG, Chilongola JO. 2025. Deploying metagenomics to characterize microbial pathogens during outbreak of acute febrile illness among children in Tanzania. Pathogens 14:601. doi:10.3390/pathogens14060601 PMC12196098 40559609

[40] Oechslin CP, Lenz N, Liechti N, Ryter S, Agyeman P, Bruggmann R, Leib SL, Beuret CM. 2018. Limited correlation of shotgun metagenomics following host depletion and routine diagnostics for viruses and bacteria in low concentrated surrogate and clinical samples. Front Cell Infect Microbiol 8:375. doi:10.3389/fcimb.2018.00375 PMC6206298 30406048

[41] Jurasz H, Pawłowski T, Perlejewski K. 2021. Contamination issue in viral metagenomics: problems, solutions, and clinical perspectives. Front Microbiol 12:745076. doi:10.3389/fmicb.2021.745076 PMC8564396 34745046

[42] Bailey AL. 2025. Here we go again: more diseases dubiously attributed to pegivirus infection. PLoS Pathog 21:e1013641. doi:10.1371/journal.ppat.1013641 PMC12561905 41150679

[43] Hoffmann H, Schmoldt S, Trülzsch K, Stumpf A, Bengsch S, Blankenstein T, Heesemann J, Roggenkamp A. 2005. Nosocomial urosepsis caused by *Enterobacter kobei* with aberrant phenotype. Diagn Microbiol Infect Dis 53:143–147. doi:10.1016/j.diagmicrobio.2005.06.008 16168611

[44] Manandhar S, Nguyen Q, Pham DT, Amatya P, Rabaa M, Dongol S, Basnyat B, Dixit SM, Baker S, Karkey A. 2022. A fatal outbreak of neonatal sepsis caused by *mcr-10*-carrying *Enterobacter kobei* in a tertiary care hospital in Nepal. J Hosp Infect 125:60–66. doi:10.1016/j.jhin.2022.03.01535460799

[45] Reddy EA, Shaw AV, Crump JA. 2010. Community-acquired bloodstream infections in Africa: a systematic review and meta-analysis. Lancet Infect Dis 10:417–432. doi:10.1016/S1473-3099(10)70072-4 PMC3168734 20510282

[46] Saha S, Ramesh A, Kalantar K, Malaker R, Hasanuzzaman M, Khan LM, Mayday MY, Sajib MSI, Li LM, Langelier C, Rahman H, Crawford ED, Tato CM, Islam M, Juan Y-F, Bourcy C, Dimitrov B, Wang J, Tang J, Sheu J, Egger R, Carvalho TR, Wilson MR, Saha SK, DeRisi JL. 2019. Unbiased metagenomic sequencing for pediatric meningitis in Bangladesh reveals neuroinvasive chikungunya virus outbreak and other unrealized pathogens. mBio 10:e02877-19. doi:10.1128/mbio.02877-19 PMC6918088 31848287

[47] Ajogbasile FV, Oguzie JU, Oluniyi PE, Eromon PE, Uwanibe JN, Mehta SB, Siddle KJ, Odia I, Winnicki SM, Akpede N, Akpede G, Okogbenin S, Ogbaini-Emovon E, MacInnis BL, Folarin OA, Modjarrad K, Schaffner SF, Tomori O, Ihekweazu C, Sabeti PC, Happi CT. 2020. Real-time metagenomic analysis of undiagnosed fever cases unveils a yellow fever outbreak in Edo State, Nigeria. Sci Rep 10:3180. doi:10.1038/s41598-020-59880-w PMC7035389 32081931

[48] Onoja BA, Oguzie JU, George UE, Asoh KE, Ajayi P, Omofaye TF, Igeleke IO, Eromon P, Harouna S, Parker E, Adeniji AJ, Happi CT. 2024. Whole genome sequencing unravels cryptic circulation of divergent dengue virus lineages in the rainforest region of Nigeria. Emerg Microbes Infect 13:2307511. doi:10.1080/22221751.2024.2307511 PMC10829817 38240324

[49] Quick J, Grubaugh ND, Pullan ST, Claro IM, Smith AD, Gangavarapu K, Oliveira G, Robles-Sikisaka R, Rogers TF, Beutler NA, et al.. 2017. Multiplex PCR method for MinION and Illumina sequencing of Zika and other virus genomes directly from clinical samples. Nat Protoc 12:1261–1276. doi:10.1038/nprot.2017.066 PMC5902022 28538739

[50] Vogels CBF, Hill V, Breban MI, Chaguza C, Paul LM, Sodeinde A, Taylor-Salmon E, Ott IM, Petrone ME, Dijk D, et al.. 2024. DengueSeq: a pan-serotype whole genome amplicon sequencing protocol for dengue virus. BMC Genomics 25:433. doi:10.1186/s12864-024-10350-x PMC11062901 38693476

